# Using Enhanced Russell Model to Solve Inverse Data Envelopment Analysis Problems

**DOI:** 10.1155/2014/571896

**Published:** 2014-05-05

**Authors:** G. R. Jahanshahloo, F. Hosseinzadeh Lotfi, M. Rostamy-Malkhalifeh, S. Ghobadi

**Affiliations:** Department of Mathematics, Islamic Azad University, Science and Research Branch, Tehran, Iran

## Abstract

This paper studies the inverse data envelopment analysis using the nonradial enhanced Russell model. Necessary and sufficient conditions for inputs/outputs determination are introduced based on Pareto solutions of multiple-objective linear programming. In addition, an approach is investigated to identify extra input/lack output in each of input/output components (maximum/minimum reduction/increase amounts in each a of input/output components). In addition, the following question is addressed: if among a group of DMUs, it is required to increase inputs and outputs to a particular unit and assume that the DMU maintains its current efficiency level with respect to other DMUs, how much should the inputs and outputs of the DMU increase? This question is discussed as inverse data envelopment analysis problems, and a technique is suggested to answer this question. Necessary and sufficient conditions are established by employing Pareto solutions of multiple-objective linear programming as well.

## 1. Introduction

Data envelopment analysis (DEA) is a nonparametric method based on linear programming for computing relative efficiencies of a decision making unit (DMU) by comparing it with other DMUs such that they produce the same multiple outputs by consuming the same multiple inputs. This method was first introduced by Charnes et al. (CCR model) [1] and extended by Banker et al. (BCC model) [2]. In the two recent decades, a wide range of research in operations research field has been allocated to this technique; see, for example, [3–5].

Relationships between DEA and MOLP can be applied as instruments in strategic planning and management control. These two types of models have much in common. However, DEA is to assess past performances as part of the management control function while MOLP is to plan future performances. These relationships have been used and developed by many scholars; see, for example, [6–12].

Inverse DEA is one of the most noticeable subjects both practically and theoretically. This concept first was discussed by Zhang and Cui [13]. Some of the questions are introduced by Wei et al. [14] in inverse DEA filed. They considered inverse DEA to answer this question: “if among a group of DMUs, we increase certain inputs to a particular unit and assume that the DMU maintains its current efficiency level with respect to other DMUs, how much should the outputs of the DMU increase?” In order to answer this question, Wei et al. [14] and Yan et al. [15] offered a linear programming problem to estimate the outputs when the unit under assessment is weakly efficient and a MOLP model when the unit under assessment is inefficient.

Moreover, in the following, Hadi-Vencheh et al. [16, 17] attempted to answer this question: “if among a group of DMUs, we increase certain outputs to a particular unit and assume that the DMU maintains its current efficiency level with respect to other DMUs, how much should the inputs of the DMU increase?” In their studies, the proposed models by Wei et al. [14] have been developed. After introducing inverse DEA, some of researchers studied it theoretically and practically [15–21].

In all investigations done on inverse DEA, researchers considered radial models such as CCR [1], BCC [2], ST [22], and FG [23] models. As it is known, the nonradial models have some different properties compared with the radial models. Therefore, in some cases, answering the questions presented in the literature inverse DEA with the nonradial models can provide more appropriate information. Consequently, for more suitable analysis, one of the nonradial models, for instance, additive models or slack-based models, can be considered. In this research, to solve some of the above problems, the nonradial Enhanced Russell Model (ERM) [24] is considered. In addition, a new problem in inverse DEA field is introduced: “if among a group of DMUs for a particular unit, the decision maker is required to increase inputs and outputs, in which, the DMU maintains its current efficiency level with respect to other DMUs, how much should the inputs and outputs of the DMU increase?” Pareto solutions of MOLP are used to solve this problem.

The paper is organized as follows. In Section 2, DEA models are reviewed and extended and the problem is stated in DEA terms. It is shown that how the inverse DEA problem (increment of the outputs) can be converted to and solved by a multiobjective programming problem, when the DMU is ERM efficient. If there exists a lack in each of the output components, its amount is specified. In Section 3, the question proposed by Wei et al. [14] is answered, when the DMU is ERM efficient. Likewise, if there exists an extra in each of the input components, its amount is specified. The new problem is solved in Section 4. For a special decision making unit providing that the ERM-efficiency score remains unchanged, necessary and sufficient conditions are introduced based on Pareto solutions of multiple-objective linear programming problems to find the minimum and maximum increase of input and output vectors, respectively. In Section 5, two examples are used to illustrate our calculation method. Finally, Section 6 demonstrates some conclusions and suggestions for future research.

## 2. Estimate Inputs

Let us consider a set of  DMU_*s*_{DMU_*j*_ : *j* = 1,…, *n*}, in which DMU_*j*_ produces multiple outputs *y*_*rj*_  (*r* = 1,…, *s*), by utilizing multiple inputs *x*_*ij*_  (*i* = 1,…, *m*). Let input and output for DMU_*j*_ be denoted by *X*_*j*_ = (*x*_1*j*_,*x*_2*j*_,…,*x*_*mj*_)^*t*^ and *Y*_*j*_ = (*y*_1*j*_, *y*_2*j*_,…,*y*_*sj*_)^*t*^, respectively. Also, suppose *X*_*j*_≩0 and *Y*_*j*_≩0. The nonradial enhanced Russell model (ERM) [24] is considered for measuring the relative efficiency of the unit under assessment DMU_*o*_, *o* ∈ {1,2,…, *n*}, as follows:

(1) ρo∗=min⁡ (1/m)∑i=1mθi(1/s)∑r=1sφr s.t.   ∑j=1nλjxij≤θixio, i=1,…,m∑j=1nλjyrj≥φryro, r=1,…,sθi≤1, φr≥1, i=1,…,m,  r=1,…,s,λ∈Ω,

where

(2) Ω={λ ∣ λ=(λ1,…,λn),δ1(∑j=1nλj+δ2(−1)δ3ν)=δ1, ν≥0, λj≥0, j=1,…,n}.

Here *δ*_1_, *δ*_2_, and *δ*_3_ are parameters with 0-1 values. It is obvious that if *δ*_1_ = 0, then model (1) is under constant returns to scale (CRC), if *δ*_1_ = 1 and *δ*_2_ = 0, then model (1) is under variable returns to scale (VRS), if *δ*_1_ = *δ*_2_ = 1 and *δ*_3_ = 0, then model (1) is under a nonincreasing returns to scale (NIRS), and if *δ*_1_ = *δ*_2_ = *δ*_3_ = 1, then model (1) is under a nondecreasing returns to scale (NDRS) assumption of the production technology.

**Remark 1**

Although in this study under discussion results satisfy CRC, VRS, NIRS, and NDRS assumption of the production technology, but the CRC model is considered only for simplicity.

**Definition 2 (ERM-efficiency, see [24])**

The optimal value *ρ*_o_* of the model (1) is called the ERM-efficiency score of DMU_*o*_. DMU_*o*_ is ERM efficient, if and only if *ρ*_o_* = 1 (this condition is equivalent to *θ*_i_* = 1 and *φ*_r_* = 1 for each *i* = 1,…, *m*, *r* = 1,…, *s* in any optimal solution).

In [13] Zhang and Cui introduced inverse DEA. Since then this problem has allocated to itself some of researches in DEA field; see, for example [5, 14, 16–19, 21]. Based upon investigated results by Hadi-Vencheh et al. [16], this question is considered: suppose that the DMU_*o*_ is ERM efficient, if the ERM-efficiency score of DMU_*o*_ remains unchanged, but the outputs increase, how much should the inputs of the DMU_*o*_ increase? 

To answer the above question, until the end of this section, presume that the outputs of DMU_*o*_ are increased from *Y*_*o*_ to *β*_*o*_ = *Y*_*o*_ + Δ*Y*_*o*_, where Δ*Y*_*o*_≩0. The objective of the problem is to estimate the input vector on the condition that DMU_*o*_ is still ERM efficient. In fact,

(3) $$\begin{array}{c} \alpha_{o}^{*}={\left(\alpha_{1o}^{*},\alpha_{2o}^{*},\ldots ,\alpha_{mo}^{*}\right)}^{t}=X_{o}+\Delta X_{o},\;\Delta X_{o}≩0. \end{array}$$

Assume DMU_*n*+1_ represents DMU_*o*_ after modification of the inputs and outputs. The following model is considered to calculate the ERM efficiency of DMU_*n*+1_:

(4) ρo+∗=min⁡ (1/m)∑i=1mθi(1/s)∑r=1sφrs.t.  ∑j=1nλjxij+αio∗λn+1≤θiαio∗, i=1,…,m      ∑j=1nλjyrj+βroλn+1≥φrβro, r=1,…,s      θi≤1, φr≥1, i=1,…,m, r=1,…,s      λj≥0, j=1,…,n+1.

Along with [16], the following definition is considered.

**Definition 3**

If the optimal values of models (1) and (4) are equal, it is said that the ERM efficiency remains unchanged; that is, eff(*α*_o_*, *β*_*o*_) = eff(*X*_*o*_, *Y*_*o*_).

To answer the above question, based on the results of [16], the following MOLP model is considered:

(5) min⁡ (α1o,…,αmo) s.t.  ∑j=1nλjxij≤θi∗αio, i=1,…,m    ∑j=1nλjyrj≥φr∗βro, r=1,…,s    αio≥xio, i=1,…,m    λj≥0, j=1,…,n,

where (θ* = (*θ*_1_*,…, *θ*_m_*), φ* = (*φ*_1_*,…, *φ*_s_*)) is an optimal solution to problem (1).

**Definition 4 (see [16])**

Let (λ*, *α*_o_*) be a feasible solution to problem (5). If there is no feasible solution (*λ*, *α*_*o*_) of (5) such that *α*_*io*_ ≤ *α*_io_* for all *i* = 1,…, *m* and *α*_*io*_ < *α*_io_* for at least one *i*, then it is said the (λ*, *α*_o_*) is a Pareto (strongly efficient) solution to problem (5).

**Definition 5 (see [14])**

Let (λ*, *α*_o_*) be a feasible solution to problem (5). If there is no feasible solution (*λ*, *α*_*o*_) of (5) such that *α*_*io*_ < *α*_io_* for all *i* = 1,…, *m*, then it is said the (λ*, *α*_o_*) is a weakly Pareto (efficient) solution to problem (5).

**Theorem 6**

Suppose that the *DMU*_*o*_ is *ERM* efficient and (λ*, θ*, φ*) is an optimal solution to problem (1) (*θ*_i_* = 1, *φ*_r_* = 1 for all *i*, *r* and *ρ*_o_* = 1). Let $\left({\hat{\lambda }}^{*},{\hat{\alpha }}_{o}^{*}\right)$ be a Pareto solution to problem (5) such that ${\hat{\alpha }}_{o}^{*}>X_{o}$ , and (*λ* ^+∗^, *θ* ^+∗^, *φ* ^+∗^) is an optimal solution to problem (4) with the optimal value of *ρ*_*o*_^+∗^. In addition, suppose that the optimal value and an optimal solution of the problem

(6) Ko∗=max⁡ 1s∑r=1skro   
s.t.  ∑j=1n(λj+∗+λn+1+∗λ^j∗)yrj≥kroφr∗βro=kroβro,r=1,…,s      kro≥1, r=1,…,s

are *K*_o_* and *k*_o_* = (*k*_1o_*,…, *k*_so_*), respectively. If the inputs of the *DMU*_*o*_ are increased to ${\hat{\alpha }}_{o}^{*}$ , then

- if *K*_o_* = 1 (it is clear *k*_ro_* = 1, for each *r*), then $eff({\hat{\alpha }}_{o}^{*},\beta_{o})=eff(X_{o},Y_{o})$ ,
- if *K*_o_* > 1, then $eff({\hat{\alpha }}_{o}^{*},k_{o}^{*}⋆\beta_{o})=eff(X_{o},Y_{o})$ .

**Remark 7**

*k*_o_*⋆*β*_*o*_ = (*k*_1o_*β_1*o*_,…, *k*_so_*β_*so*_) and (*k*_o_*⋆*β*_*o*_) − *β*_*o*_ = (*k*_1o_*β_1*o*_ − *β*_1*o*_,…, *k*_so_*β_*so*_ − *β*_*so*_). Note that the *k*_ro_*β_*ro*_ − *β*_*ro*_ indicates the lack-output amount in *r*th output component of the DMU_*o*_. In other words, for the decision maker to preserve the ERM-efficiency score of the DMU_*o*_ while the inputs increase from *X*_*o*_ to ${\hat{\alpha }}_{o}^{*}$ , they are required to increase the outputs from *Y*_*o*_ to *k*_o_*⋆*β*_*o*_.

**Proof**

Because $\left({\hat{\lambda }}^{*},{\hat{\alpha }}_{o}^{*}\right)$ is a feasible solution of (5), the following inequalities are held:

(7) $$\begin{array}{c} \overset{n}{\underset{j=1}{\sum }}{\hat{\lambda }}_{j}^{*}x_{ij}\leq \theta_{i}^{*}{\hat{\alpha }}_{io}^{*}={\hat{\alpha }}_{io}^{*},\;i=1,\ldots ,m \end{array}$$

(8) $$\begin{array}{c} \overset{n}{\underset{j=1}{\sum }}{\hat{\lambda }}_{j}^{*}y_{rj}\geq \varphi_{r}^{*}\beta_{ro}=\beta_{ro},\;r=1,\ldots ,s \end{array}$$

(9) $$\begin{array}{c} {\hat{\alpha }}_{io}^{*}\geq x_{io},\;i=1,\ldots ,m \end{array}$$

(10) $$\begin{array}{c} {\hat{\lambda }}_{j}^{*}\geq 0,\;j=1,\ldots ,n. \end{array}$$

With regard to inequalities (7), (8), and (10) it is obvious that $\left(\overset{-}{\lambda },\theta^{*},\varphi^{*}\right)$ is a feasible solution to problem (4), where $\overset{-}{\lambda }=({\hat{\lambda }}^{*},0)\geq 0$ , θ* = (*θ*_1_*,…, *θ*_m_*) ∈ *R*_+_^*m*^, and φ* = (*φ*_1_*,…, *φ*_s_*) ∈ *R*_+_^*s*^, therefore, *ρ*_*o*_^+∗^ ≤ *ρ*_o_*.

Using inequalities (7) and (8) in problem (4), the following results are obtained:

(11) θi+∗α^io∗≥∑j=1nλj+∗xij+λn+1+∗α^io∗=∑j=1nλj+∗xij+λn+1+∗(∑j=1nλ^j∗xij),θi+∗α^io∗≥∑j=1n(λj+∗+λn+1+∗λ^j∗)xij, i=1,…,m

(12) φr+∗βro≤∑j=1nλj+∗yrj+λn+1+∗βro≤∑j=1nλj+∗yrj+λn+1+∗(∑j=1nλ^j∗yrj),φr+∗βro≤∑j=1n(λj+∗+λn+1+∗λ^j∗)yrj, r=1,…,s.

Set ${\tilde{\lambda }}_{j}=\lambda_{j}^{+*}+\lambda_{n+1}^{+*}{\hat{\lambda }}_{j}^{*}$ for each *j* = 1,…, *n*, and it is obviously seen that $\tilde{\lambda }=({\tilde{\lambda }}_{1},\ldots ,{\tilde{\lambda }}_{n})\geq 0$ .

Now by contradiction assume that *ρ*_*o*_^+∗^ < *ρ*_o_*.

Since *ρ*_*o*_^+∗^ < *ρ*_o_*, then (1/*m*)∑_*i*=1_^*m*^ *θ*_*i*_^+∗^ < 1 or (1/*s*)∑_*r*=1_^*s*^ *φ*_*r*_^+∗^ > 1, so that there are two cases.

*(a) Let *(1/*m*)∑_*i*=1_^*m*^ *θ*_*i*_^+∗^ < 1. There exists at least one *t*, 1 ≤ *t* ≤ *m*, such that *θ*_*t*_^+∗^ < 1. With regard to inequalities (11), the following inequality is obtained:

(13) $$\begin{array}{c} \overset{n}{\underset{j=1}{\sum }}{\tilde{\lambda }}_{j}x_{tj}\leq \theta_{t}^{+*}{\hat{\alpha }}_{to}^{*}<{\hat{\alpha }}_{to}^{*}. \end{array}$$

On the other hand, since, for each *i* = 1,…, *m*, ${\hat{\alpha }}_{io}^{*}>x_{io}$ , therefore if

(14) $$\begin{array}{c} \eta_{t}=\min\left\{{\hat{\alpha }}_{to}^{*}-\overset{n}{\underset{j=1}{\sum }}{\tilde{\lambda }}_{j}x_{tj},{\hat{\alpha }}_{to}^{*}-x_{to}\right\}, \end{array}$$

then *η*_*t*_ > 0. Now define

(15) $$\begin{array}{c} {\tilde{\alpha }}_{io}=\{\begin{array}{cc} {\hat{\alpha }}_{io}^{*}, & \text{if}\;\;i\neq t,\\ {\hat{\alpha }}_{io}^{*}-\eta_{t}, & \text{if}\;\;i=t. \end{array} \end{array}$$

Taking (14) into consideration, the following inequalities are obtained:

(16) $$\begin{array}{c} \eta_{t}\leq {\hat{\alpha }}_{to}^{*}-\overset{n}{\underset{j=1}{\sum }}{\tilde{\lambda }}_{j}x_{tj}⟹\overset{n}{\underset{j=1}{\sum }}{\tilde{\lambda }}_{j}x_{tj}\leq {\hat{\alpha }}_{to}^{*}-\eta_{t}=\theta_{t}^{*}{\tilde{\alpha }}_{to}, \end{array}$$

(17) $$\begin{array}{c} \eta_{t}\leq {\hat{\alpha }}_{to}^{*}-x_{to}⟹x_{to}\leq {\hat{\alpha }}_{to}^{*}-\eta_{t},⟹x_{to}\leq {\tilde{\alpha }}_{to}, \end{array}$$

which implies that ${\tilde{\alpha }}_{o}\geq X_{o}$ .

Based on $\tilde{\lambda }\geq 0$ and (11), (12), and (16) and ${\tilde{\alpha }}_{o}\geq X_{o}$ , $\left(\tilde{\lambda },{\tilde{\alpha }}_{o}\right)$ is a feasible solution to problem (5), where ${\tilde{\alpha }}_{io}\leq {\hat{\alpha }}_{io}^{*}$ for all *i* = 1,…, *m*, and ${\tilde{\alpha }}_{io}<{\hat{\alpha }}_{io}^{*}$ for some *i* = 1,…, *m*. This contradicts the assumption that $\left({\hat{\lambda }}^{*},{\hat{\alpha }}_{o}^{*}\right)$ is a Pareto solution to problem (5).

*(b) Let *(1/*s*)∑_*r*=1_^*s*^ *φ*_*r*_^+∗^ > 1. There exists at least one *t*, 1 ≤ *t* ≤ *s*, such that *φ*_*t*_^+∗^ > 1. By (12), the following strict inequality is obtained:

(18) $$\begin{array}{c} \overset{n}{\underset{j=1}{\sum }}{\tilde{\lambda }}_{j}y_{tj}\geq \varphi_{t}^{+*}\beta_{to}>\beta_{to} \end{array}$$

and, therefore, there exists a *μ*_*t*_ > 1 that satisfies

(19) $$\begin{array}{c} \overset{n}{\underset{j=1}{\sum }}{\tilde{\lambda }}_{j}y_{tj}\geq \mu_{t}\beta_{to}=\left(\mu_{t}k_{to}^{*}\right)\beta_{to}. \end{array}$$

Now define

(20) $$\begin{array}{c} {\tilde{k}}_{ro}=\{\begin{array}{cc} k_{ro}^{*}=1, & \text{if}\;\;r\neq t,\\ k_{ro}^{*}\mu_{t}=\mu_{t}, & \text{if}\;\;r=t. \end{array} \end{array}$$

It is obvious that ${\tilde{k}}_{o}=({\tilde{k}}_{1o},\ldots ,{\tilde{k}}_{so})$ is a feasible solution to problem (6), such that

(21) $$\begin{array}{c} K_{o}^{*}=\frac{1}{s}\overset{s}{\underset{r=1}{\sum }}k_{ro}<\frac{1}{s}\overset{s}{\underset{r=1}{\sum }}{\tilde{k}}_{ro}={\tilde{K}}_{o}. \end{array}$$

This contradicts the assumption expressing that the optimal value of problem (6) is *K*_o_*. Consequently, in each case *ρ*_*o*_^+∗^≮*ρ*_o_*, and since *ρ*_*o*_^+∗^ ⩽ *ρ*_o_*, therefore, *ρ*_*o*_^+∗^ = *ρ*_o_*.

(ii) with replacing the notation *β*_*ro*_ by *k*_ro_*β_*ro*_ for each *r* = 1,…, *s*, in problem (6), clearly, optimal value of problem (6) is 1. Therefore, according to part (i), then $\text{eff}({\hat{\alpha }}_{o}^{*},k_{o}^{*}⋆\beta_{o})=\text{eff}(X_{o},Y_{o})$ .

Although Theorem 6 satisfies for all units that be ERM-efficient, but the following theorem is the converse version of Theorem 6 that satisfies for all DMU_*s*_.

**Theorem 8**

Suppose that the (λ*, θ*, φ*) is an optimal solution to problem (1) with the optimal value of *ρ*_o_*. Let $\left(\overset{-}{\lambda },{\overset{-}{\alpha }}_{o}\right)$ be a feasible solution to problem (5). If $eff({\overset{-}{\alpha }}_{o},\beta_{o})=eff(X_{o},Y_{o})$ , then $\left(\overset{-}{\lambda },{\overset{-}{\alpha }}_{o}\right)$ must be a Pareto solution to problem (5).

**Proof**

If $\left(\overset{-}{\lambda },{\overset{-}{\alpha }}_{o}\right)$ is not a Pareto solution to problem (5), then there would exist another feasible solution of (5), $\left(\tilde{\lambda },{\tilde{\alpha }}_{o}\right)$ in which, ${\tilde{\alpha }}_{io}\leq {\overset{-}{\alpha }}_{io}$ for *i* = 1,…, *m* and ${\tilde{\alpha }}_{io}<{\overset{-}{\alpha }}_{io}$ for at least one *i* ∈ { 1,…, *m*}. Let $I=\left\{i|{\tilde{\alpha }}_{io}<{\overset{-}{\alpha }}_{io}\right\}$ . Then

(22) $$\begin{array}{c} \overset{n}{\underset{j=1}{\sum }}{\tilde{\lambda }}_{j}x_{ij}\leq \theta_{i}^{*}{\tilde{\alpha }}_{io}<\theta_{i}^{*}{\overset{-}{\alpha }}_{io},\;i\in I, \end{array}$$

(23) $$\begin{array}{c} \overset{n}{\underset{j=1}{\sum }}{\tilde{\lambda }}_{j}x_{ij}\leq \theta_{i}^{*}{\tilde{\alpha }}_{io}\leq \theta_{i}^{*}{\overset{-}{\alpha }}_{io},\;i\notin I, \end{array}$$

(24) $$\begin{array}{c} \overset{n}{\underset{j=1}{\sum }}{\tilde{\lambda }}_{j}y_{rj}\geq \varphi_{r}^{*}\beta_{ro},\;r=1,\ldots ,s, \end{array}$$

(25) $$\begin{array}{c} {\tilde{\lambda }}_{j}\geq 0,\;j=1,\ldots ,n. \end{array}$$

By inequality (22) there exists *μ*_*i*_ < 1 for all *i* ∈ *I*, such that

(26) $$\begin{array}{c} \overset{n}{\underset{j=1}{\sum }}{\tilde{\lambda }}_{j}x_{ij}\leq \left(\mu_{i}\theta_{i}^{*}\right){\overset{-}{\alpha }}_{io},\;i\in I. \end{array}$$

For each *i* = 1,…, *m* and *r* = 1,…, *s*, define

(27) $$\begin{array}{c} {\overset{-}{\theta }}_{i}=\{\begin{array}{cc} \mu_{i}\theta_{i}^{*} & \text{if}\;\;i\in I,\\ \theta_{i}^{*}, & \text{if}\;\;i\notin I, \end{array}\;\;{\overset{-}{\varphi }}_{r}=\varphi_{r}^{*},\;r=1,\ldots ,s. \end{array}$$

Based on inequalities (23)–(26), $\left(\tilde{\lambda },\overset{-}{\theta },\overset{-}{\varphi }\right)$ is a feasible solution to problem (1) (only restrictions on the right have replaced the *X*_*o*_ and *Y*_*o*_ by ${\overset{-}{\alpha }}_{o}$ and *β*_*o*_ in (1), resp.), where $\overset{-}{\theta }=({\overset{-}{\theta }}_{1},\ldots ,{\overset{-}{\theta }}_{m})$ and $\overset{-}{\varphi }=({\overset{-}{\varphi }}_{1},\ldots ,{\overset{-}{\varphi }}_{s})$ . Therefore

(28) $$\begin{array}{c} {\overset{-}{\rho }}_{o}=\frac{\left(1/m\right)\sum_{i=1}^{m}{\overset{-}{\theta }}_{i}}{\left(1/s\right)\sum_{r=1}^{s}{\overset{-}{\varphi }}_{r}}<\frac{\left(1/m\right)\sum_{i=1}^{m}\theta_{i}^{*}}{\left(1/s\right)\sum_{r=1}^{s}\varphi_{r}^{*}}=\rho_{o}^{*}, \end{array}$$

which is against the assumption expressing that $\text{eff}({\overset{-}{\alpha }}_{o},\beta )=\rho_{o}^{*}$ .

Along with Theorem 2 in Section 5 in [14] the following theorem is considered.

**Theorem 9**

Suppose that the *DMU*_*o*_ is *ERM* efficient and the inputs and the outputs for *DMU*_*o*_ are going to increase from *X*_*o*_ and *Y*_*o*_ to *α*_*o*_ = *X*_*o*_ + Δ*X*_*o*_ and *β*_*o*_ = *Y*_*o*_ + Δ*Y*_*o*_, respectively, where there are Δ*X*_*o*_≩0, Δ*Y*_*o*_≩0. It is worth mentioning that it is required that the (*α*_*o*_, *β*_*o*_) belongs to the production possibility set. Consider the following problem:

(29) ρ^o=min⁡ (1/m)∑i=1mθi(1/s)∑r=1sφr   
s.t.  ∑j=1nλjxij≤θiαio, i=1,…,m     ∑j=1nλjyrj≥φrβro, r=1,…,s     θi≤1, φr≥1, i=1,…,m, r=1,…,s     λj≥0, j=1,…,n.

If the inputs and outputs of *DMU*_*o*_ are increased from *X*_*o*_ to ${\hat{X}}_{o}=({\hat{\theta }}_{1}x_{1o},\ldots ,{\hat{\theta }}_{m}x_{mo})$ and ${\hat{Y}}_{o}=({\hat{\varphi }}_{1}y_{1o},\ldots ,{\hat{\varphi }}_{s}y_{so})$ , respectively, and $\left(\hat{\lambda },\hat{\theta },\hat{\varphi }\right)$ is an optimal solution to problem (29) with the optimal value of $\hat{\rho }$ , then $eff(X_{o},Y_{o})=eff({\hat{X}}_{o},{\hat{Y}}_{o})$ .

**Proof**

When the inputs and outputs of the DMU_*o*_ convert to ${\hat{X}}_{o}$ and ${\hat{Y}}_{o}$ , respectively, the ERM-efficiency score of the DMU equals the optimal value of the problem below

(30) ρ~o=min⁡ (1/m)∑i=1mθi(1/s)∑r=1sφr   s.t.  ∑j=1nλjxij+λn+1x^io≤θix^io, i=1,…,m     ∑j=1nλjyrj+λn+1y^ro≥φry^ro, r=1,…,s     θi≤1, φr≥1, i=1,…,m, r=1,…,s     λj≥0, j=1,…,n+1.

Suppose that $\left(\tilde{\lambda },\tilde{\theta },\tilde{\varphi }\right)$ is an optimal solution to problem (30) with the optimal value of ${\tilde{\rho }}_{o}$ . To prove the theorem, ${\tilde{\rho }}_{o}=1$ should be shown. By contradiction assume that ${\tilde{\rho }}_{o}<1$ .

Problem (30) is nonlinear, but it can be converted to the following linear programming problem by employing the *t* = 1/(1/*s*)∑_*r*=1_^*s*^ *φ*_*r*_ > 0:

(31) ρ~−o=min⁡ 1m∑i=1mθ−i   s.t.  ∑j=1nλ−jxij+λ−n+1x^io≤θ−ix^io, i=1,…,m     ∑j=1nλ−jyrj+λ−n+1y^ro≥φ−ry^ro, r=1,…,s     ∑r=1sφ−r=s, t≥ϵ,     θ−i≤t, φ−r≥t, i=1,…,m, r=1,…,s     λ−j≥0, j=1,…,n+1,

where $\left(\overset{-}{\theta },\overset{-}{\varphi })=(t\theta ,t\varphi \right)$ , ${\overset{-}{\lambda }}_{j}=t\lambda_{j}$ for *j* = 1,…, *n*, and *ϵ* is non-Archimedean infinitesimal. Note that *t* ≥ *ϵ* is a redundant constraint in problem (31) and hence can be omitted. The dual problem (31) is as follows:

(32)  max⁡ sz  s.t.  UtYj−VtXj≤0, j=1,…,n    UtY^o−VtX^o≤0,

(33)     vix^io−τi=1m, i=1,…,m    −ury^ro+wr+z=0, r=1,…,s    V≥0, U≥0, τ≥0, z∈R,

(34)     emτ−esW=0,    V≥0, U≥0, τ≥0, z∈R,

where *e*_*m*_ = (1,…, 1) ∈ *R* ^*m*^ and *e*_*s*_ = (1,…, 1) ∈ *R* ^*s*^. Assume $\left(\overset{-}{V},\overset{-}{U},\overset{-}{\tau },\overset{-}{W},\overset{-}{z}\right)$ is an optimal solution to the above problem. Based on the duality Theorem, the following relation is held:

(35) $$\begin{array}{c} s\overset{-}{z}={\tilde{\rho }}_{o}<1. \end{array}$$

Also, constraint sets (33) are added together and result in

(36) $$\begin{array}{c} -{\overset{-}{U}}^{t}{\hat{Y}}_{o}+e_{s}\overset{-}{W}+s\overset{-}{z}+{\overset{-}{V}}^{t}{\hat{X}}_{o}-e_{m}\overset{-}{\tau }=1; \end{array}$$

taking (35) into consideration, the following inequality is obtained:

(37) $$\begin{array}{c} s\overset{-}{z}={\tilde{\rho }}_{o}<1=-{\overset{-}{U}}^{t}{\hat{Y}}_{o}+e_{s}\overset{-}{W}+s\overset{-}{z}+{\overset{-}{V}}^{t}{\hat{X}}_{o}-e_{m}\overset{-}{\tau }, \end{array}$$

and, therefore

(38) $$\begin{array}{c} {\overset{-}{U}}^{t}{\hat{Y}}_{o}-{\overset{-}{V}}^{t}X_{o}<0. \end{array}$$

Since the variable ${\overset{-}{\lambda }}_{n+1}$ is corresponding to the constraint (32) in the above problem that is unbinding at each optimal solution, therefore, according to the complementary slackness theorem, ${\overset{-}{\lambda }}_{n+1}$ must be 0, and so *λ*_*n*+1_ = 0.

Consequently, ${\tilde{\rho }}_{o}$ is also the optimal value of the following problem:

(39) ρ~o=min⁡ (1/m)∑i=1mθi(1/s)∑r=1sφr   s.t.  ∑j=1nλjxij≤θix^io, i=1,…,m     ∑j=1nλjyrj≥φry^ro, r=1,…,s     θi≤1, φr≥1, i=1,…,m, r=1,…,s     λj≥0, j=1,…,n.

Now, consider the following problem:

(40) ρ−o=min⁡ (1/m)∑i=1mθ^iθi(1/s)∑r=1sφ^rφr  s.t.  ∑j=1nλjxij≤θ^iθixio, i=1,…,m     ∑j=1nλjyrj≥φ^rφryro, r=1,…,s     θ^iθi≤1, φ^rφr≥1, i=1,…,m,r=1,…,s     λj≥0, j=1,…,n.

Clearly, $\left(\tilde{\lambda },\tilde{\theta },\tilde{\varphi }\right)$ is a feasible solution to problem (40), and so

(41) $$\begin{array}{c} {\overset{-}{\rho }}_{o}\leq \frac{\left(1/m\right)\sum_{i=1}^{m}{\hat{\theta }}_{i}{\tilde{\theta }}_{i}}{\left(1/s\right)\sum_{r=1}^{s}{\hat{\varphi }}_{r}{\tilde{\varphi }}_{r}}. \end{array}$$

On the other hand, since ${\tilde{\rho }}_{o}<1$ , there exists at least one *t*, 1 ≤ *t* ≤ *m* or 1 ≤ *t* ≤ *s*, such that ${\tilde{\theta }}_{t}<1$ , or ${\tilde{\varphi }}_{t}>1$ . Therefore, in each case

(42) $$\begin{array}{c} {\overset{-}{\rho }}_{o}\leq \frac{\left(1/m\right)\sum_{i=1}^{m}{\hat{\theta }}_{i}{\tilde{\theta }}_{i}}{\left(1/s\right)\sum_{r=1}^{s}{\hat{\varphi }}_{r}{\tilde{\varphi }}_{r}}<\frac{\left(1/m\right)\sum_{i=1}^{m}{\hat{\theta }}_{i}}{\left(1/s\right)\sum_{r=1}^{s}{\hat{\varphi }}_{r}}={\hat{\rho }}_{o}. \end{array}$$

Obviously, problem (40) is just problem (29) (replacing the notation ${\hat{\theta }}_{i}\theta_{i}$ by *θ*_*i*_ and ${\hat{\varphi }}_{r}\varphi_{r}$ by *φ*_*r*_). Therefore, in each case, the optimal value of problem (29) would be ${\overset{-}{\rho }}_{o}<{\hat{\rho }}_{o}$ , which contradicts the fact that the maximum value of (29) is ${\hat{\rho }}_{o}$ .

## 3. Estimate Outputs

In this section, the problem provided by Wei et al. [14] is considered. Based on the results of their study, this question is addressed: suppose that the DMU_*o*_ is ERM efficient, if the ERM-efficiency score of DMU_*o*_ remains unchanged while the inputs increase, and how much should the outputs of the DMU_*o*_ increase?

To solve the above problem, to the end of this section, presume that the inputs of DMU_*o*_ are increased from *X*_*o*_ to *α*_*o*_ = *X*_*o*_ + Δ*X*_*o*_, where Δ*X*_*o*_≩0. The aim of the problem is to estimate the output vector provided that the DMU_*o*_ is still ERM efficient. In fact,

(43) $$\begin{array}{c} \beta_{o}^{*}={\left(\beta_{1o}^{*},\beta_{2o}^{*},\ldots ,\beta_{so}^{*}\right)}^{t}=Y_{o}+\Delta Y_{o},\;\Delta Y_{o}≩0. \end{array}$$

Assume DMU_*n*+1_ represents DMU_*o*_ after modification of the inputs and outputs. Along the line of [14], the following model is considered to calculate the ERM efficiency of DMU_*n*+1_:

(44) ρo+∗=min⁡ (1/m)∑i=1mθi(1/s)∑r=1sφr   s.t.   ∑j=1nλjxij+αioλn+1≤θiαio, i=1,…,m      ∑j=1nλjyrj+βro∗λn+1≥φrβro∗, r=1,…,s      θi≤1, φr≥1, i=1,…,m, r=1,…,s      λj≥0, j=1,…,n+1.

Based on Definition 3 it is said that the ERM efficiency remains unchanged, if and only if eff(*α*_*o*_, *β*_o_*) = eff(*X*_*o*_, *Y*_*o*_). To answer the above question, along the line of [14], the following MOLP model is considered:

(45) max⁡ (β1o,…,βso) s.t.  ∑j=1nλjxij≤θi∗αio, i=1,…,m    ∑j=1nλjyrj≥φr∗βro, r=1,…,s    αio≥xio, i=1,…,m    λj≥0, j=1,…,n,

where (θ*, φ*) is an optimal solution to problem (1).

**Theorem 10**

Suppose that the DMU_*o*_ is ERM efficient and (λ*, θ*, φ*) is an optimal solution to problem (1). Let $\left({\hat{\lambda }}^{*},{\hat{\beta }}_{o}^{*}\right)$ be a Pareto solution to problem (45) such that ${\hat{\beta }}_{o}^{*}>Y_{o}$ , and (*λ* ^+∗^, *θ* ^+∗^, *φ* ^+∗^) is an optimal solution to problem (4) with the optimal value of *ρ*_*o*_^+∗^. Also, suppose that the optimal value and an optimal solution of the following problem:

(46) Ko∗=max⁡ 1m∑i=1mkio   
s.t.  ∑j=1n(λj+∗+λn+1+∗λ^j∗)xij≤kioθi∗αio=kioαio,i=1,…,m      kio≤1, i=1,…,m

are *K*_o_* and *k*_o_* = (*k*_1o_*,…, *k*_mo_*). If the outputs of DMU_*o*_ are increased to ${\hat{\beta }}_{o}^{*}$ , then

- if *K*_o_* = 1, then $eff(\alpha_{o},{\hat{\beta }}_{o}^{*})=eff(X_{o},Y_{o})$ ,
- if *K*_o_* < 1, then $eff(k_{o}^{*}⋆\alpha_{o},{\hat{\beta }}_{o}^{*})=eff(X_{o},Y_{o})$ .

**Remark 11**

*k*_o_*⋆*α*_*o*_ = (*k*_1o_*α_1*o*_,…, *k*_mo_*α_*mo*_) and *α*_*o*_ − (*k*_o_*⋆*α*_*o*_) = (*α*_1*o*_ − *k*_1o_*α_1*o*_,…, *α*_*mo*_ − *k*_mo_*α_*mo*_). Note that the *α*_*io*_ − *k*_io_*α_*io*_ indicates extra-input amount in *i*th input component of the DMU. In other words, for the decision maker to preserve the ERM-efficiency score of the DMU_*o*_ while the outputs increase from *Y*_*o*_ to ${\hat{\beta }}_{o}^{*}$ , they are required to increase the inputs from *X*_*o*_ to *k*_o_*⋆*α*_*o*_.

**Proof**

The proof is similar to the proof of Theorem 6.

**Theorem 12**

Assume (λ*, θ*, φ*) is an optimal solution to problem (1) with the optimal value of *ρ*_o_*. Let $\left(\overset{-}{\lambda },{\overset{-}{\beta }}_{o}\right)$ be a feasible solution to problem (45). If $eff(\alpha_{o},{\overset{-}{\beta }}_{o})=eff(X_{o},Y_{o})$ , then $\left(\overset{-}{\lambda },{\overset{-}{\beta }}_{o}\right)$ must be a Pareto solution to problem (45).

**Proof**

The proof is similar to the proof of Theorem 8.

## 4. Estimate the Minimum Increase of Inputs and the Maximum Increase of Outputs

In order to present suitable patterns to the decision maker to increase inputs and outputs for an ERM-efficient DMU, under preserving the ERM-efficiency index, this new question in field of inverse DEA is addressed: if DMU_*o*_ is ERM efficient, while the inputs and outputs are required to be increased, how much should the inputs and outputs of the DMU_*o*_ be increased? In other words, under preserving the ERM-efficiency score, how much should the minimum and maximum of input and output vectors of the DMU_*o*_ increase, respectively?

By answering the question, the decision maker may be able to take better decisions in order to extend decision making units. That is to say that the decision maker can take necessary actions by choosing a suitable strategy for spreading an ERM-efficient DMU.

The aim of addressing this question is estimating the minimum increase of inputs and the maximum increase of outputs provided that the DMU_*o*_ is still ERM efficient. In fact,

(47) $$\begin{array}{c} \alpha_{o}^{*}={\left(\alpha_{1o}^{*},\alpha_{2o}^{*},\ldots ,\alpha_{mo}^{*}\right)}^{t}=X_{o}+\Delta X_{o},\;\Delta X_{o}≩0,\\ \beta_{o}^{*}={\left(\beta_{1o}^{*},\beta_{2o}^{*},\ldots ,\beta_{so}^{*}\right)}^{t}=Y_{o}+\Delta Y_{o},\;\Delta Y_{o}≩0. \end{array}$$

**Remark 13**

Note that it is required to make the rate of increase inputs and outputs of the unit under assessment bounded; otherwise, it is possible that at least one of the components of *α*_o_* or *β*_o_* is unbounded.

Suppose that DMU_*n*+1_ represents DMU_*o*_ after modification of the inputs and outputs. Based on results of [14, 16], the following model is considered to estimate the ERM efficiency of DMU_*n*+1_:

(48) ρo+∗=min⁡ (1/m)∑i=1mθi(1/s)∑r=1sφr   s.t.  ∑j=1nλjxij+αio∗λn+1≤θiαio∗, i=1,…,m      ∑j=1nλjyrj+βro∗λn+1≥φrβro∗, r=1,…,s      θi≤1, φr≥1, i=1,…,m, r=1,…,s      λj≥0, j=1,…,n+1.

**Definition 14**

If the optimal values of problems (1) and (48) are equal, it is said that the ERM efficiency remains unchanged; that is, eff(*α*_o_*, *β*_o_*) = eff(*X*_*o*_, *Y*_*o*_).

To solve this new question, that is, to estimate the minimum increase of inputs and the maximum increase of outputs, along the line of [14, 16], the following MOLP problem is considered:

(49) min⁡ (α1o,…,αmo)max⁡ (β1o,…,βso) s.t.  ∑j=1nλjxij≤θi∗αio, i=1,…,m     ∑j=1nλjyrj≥φr∗βro, r=1,…,s     αio≥xio, i=1,…,m     βro≥yro, r=1,…,s     βo∈Γ, αo∈Λ,     λj≥0, j=1,…,n,

where θ* and φ* are an optimal solution to problem (1), Γ and Λ are bounded sets, and show the maximum rate of increase in inputs and outputs of the DMU_*o*_, respectively, such that by the decision maker are considered.

**Theorem 15**

Suppose that the *DMU*_*o*_ is ERM efficient and (λ*, θ*, φ*) is an optimal solution to problem (1). Let $\left({\hat{\lambda }}^{*},{\hat{\alpha }}_{o}^{*},{\hat{\beta }}_{o}^{*}\right)$ be a Pareto solution to problem (49) such that ${\hat{\alpha }}_{o}^{*}>X_{o},{\hat{\beta }}_{o}^{*}≩Y_{o}$ . If the inputs and outputs of DMU_*o*_ are increased to ${\hat{\alpha }}_{o}^{*}$ and ${\hat{\beta }}_{o}^{*}$ , respectively, then $eff({\hat{\alpha }}_{o}^{*},{\hat{\beta }}_{o}^{*})=eff(X_{o},Y_{o})$ .

**Proof**

Assume (*λ* ^+∗^, *θ* ^+∗^, *φ* ^+∗^) is an optimal solution to problem (48) with the optimal value of *ρ* ^+∗^. The proof is similar to the proof of Theorem 6, when replacing the notation *β*_*o*_ by ${\hat{\beta }}_{o}^{*}$ . The only difference is in case (b), that is, when (1/*s*)∑_*r*=1_^*s*^ *φ*_*r*_^+∗^ > 1. In this case, there exists at least one *p*, 1 ≤ *p* ≤ *s*, such that *φ*_*p*_^+∗^ > 1. Taking (12) into consideration, the following inequality is obtained:

(50) $$\begin{array}{c} \overset{n}{\underset{j=1}{\sum }}{\tilde{\lambda }}_{j}y_{pj}\geq \varphi_{p}^{+*}{\hat{\beta }}_{po}^{*}>{\hat{\beta }}_{po}^{*}, \end{array}$$

and, thus, there exists a *η*_*p*_ > 0 that satisfies

(51) $$\begin{array}{c} \overset{n}{\underset{j=1}{\sum }}{\tilde{\lambda }}_{j}y_{pj}\geq \left({\hat{\beta }}_{po}^{*}+\eta_{p}\right). \end{array}$$

Now, define

(52) $$\begin{array}{c} {\tilde{\beta }}_{ro}=\{\begin{array}{cc} \beta_{ro}^{*}, & \text{if}\;\;r\neq p,\\ \eta_{r}+{\hat{\beta }}_{ro}^{*}, & \text{if}\;\;r=p. \end{array} \end{array}$$

Based on $\tilde{\lambda }\geq 0$ , inequality (9), (11), and (51), and ${\tilde{\beta }}_{o}≩{\hat{\beta }}_{o}^{*}≩Y_{o}$ , $\left(\tilde{\lambda },{\hat{\alpha }}_{o}^{*},{\tilde{\beta }}_{o}\right)$ is a feasible solution to problem (49), where ${\tilde{\beta }}_{ro}\geq {\hat{\beta }}_{ro}^{*}$ for all *r* = 1,…, *s*, and ${\tilde{\beta }}_{ro}>\beta_{ro}^{*}$ for some *r* = 1,…, *s*. This contradicts the assumption expressing that the $\left({\hat{\lambda }}^{*},{\hat{\alpha }}_{o}^{*},{\hat{\beta }}_{o}^{*}\right)$ is a Pareto solution to problem (49). Therefore, in any two cases, because *ρ*_*o*_^+∗^≮*ρ*_o_* and *ρ*_*o*_^+∗^ ⩽ *ρ*_o_*, therefore *ρ*_*o*_^+∗^ = *ρ*_o_*.

**Theorem 16**

Suppose that the (λ*, θ*, φ*) is an optimal solution to problem (1) with the optimal value of *ρ*_o_*. Let $\left(\overset{-}{\lambda },{\overset{-}{\alpha }}_{o},{\overset{-}{\beta }}_{o}\right)$ be a feasible solution to the problem (49). If $eff({\overset{-}{\alpha }}_{o},{\overset{-}{\beta }}_{o})=eff(X_{o},Y_{o})$ , then $\left(\overset{-}{\lambda },{\overset{-}{\alpha }}_{o},{\overset{-}{\beta }}_{o}\right)$ must be a Pareto solution to problem (49).

**Proof**

If $\left(\overset{-}{\lambda },{\overset{-}{\alpha }}_{o},{\overset{-}{\beta }}_{o}\right)$ is not a Pareto solution to problem (49), then there would exist another feasible solution of problem (5), $\left(\tilde{\lambda },{\tilde{\alpha }}_{o},\tilde{\beta }\right)$ , such that ${\tilde{\alpha }}_{io}\leq {\overset{-}{\alpha }}_{io}$ for all *i* and ${\tilde{\beta }}_{ro}\geq {\overset{-}{\beta }}_{ro}$ for all *r*; furthermore, there exists at least one *i* in which ${\tilde{\alpha }}_{io}<{\overset{-}{\alpha }}_{io}$ or at least one *r* such that ${\tilde{\beta }}_{ro}>{\overset{-}{\beta }}_{ro}$ . Let $I=\left\{i|{\tilde{\alpha }}_{io}<{\overset{-}{\alpha }}_{io}\right\}$ and $O=\left\{r|{\tilde{\beta }}_{ro}>{\overset{-}{\beta }}_{ro}\right\}$ . Therefore

(53) $$\begin{array}{c} \overset{n}{\underset{j=1}{\sum }}{\tilde{\lambda }}_{j}x_{ij}\leq \theta_{i}^{*}{\tilde{\alpha }}_{io}<\theta_{i}^{*}{\overset{-}{\alpha }}_{io}\;i\in I \end{array}$$

(54) $$\begin{array}{c} \overset{n}{\underset{j=1}{\sum }}{\tilde{\lambda }}_{j}x_{ij}\leq \theta_{i}^{*}{\tilde{\alpha }}_{io}\leq \theta_{i}^{*}{\overset{-}{\alpha }}_{io}\;i\notin I \end{array}$$

(55) $$\begin{array}{c} \overset{n}{\underset{j=1}{\sum }}{\tilde{\lambda }}_{j}y_{rj}\geq \varphi_{r}^{*}{\tilde{\beta }}_{ro}>\varphi_{r}^{*}{\overset{-}{\beta }}_{ro}\;r\in O \end{array}$$

(56) $$\begin{array}{c} \overset{n}{\underset{j=1}{\sum }}{\tilde{\lambda }}_{j}y_{rj}\geq \varphi_{r}^{*}{\tilde{\beta }}_{ro}\geq \varphi_{r}^{*}{\overset{-}{\beta }}_{ro}\;r\notin O \end{array}$$

(57) $$\begin{array}{c} {\tilde{\lambda }}_{j}\geq 0,\;j=1,\ldots ,n. \end{array}$$

Based on inequalities (53) and (56) there exists *μ*_*i*_ < 1 for *i* ∈ *I* and *η*_*r*_ > 1 for *r* ∈ *O*, such that

(58) $$\begin{array}{c} \overset{n}{\underset{j=1}{\sum }}{\tilde{\lambda }}_{j}x_{ij}\leq \left(\mu_{i}\theta_{i}^{*}\right){\overset{-}{\alpha }}_{io},\;i\in I \end{array}$$

(59) $$\begin{array}{c} \overset{n}{\underset{j=1}{\sum }}{\tilde{\lambda }}_{j}y_{rj}\geq \left(\eta_{r}\varphi_{r}^{*}\right){\overset{-}{\beta }}_{ro},\;r\in O. \end{array}$$

For each *i* = 1,…, *n* and *r* = 1,…, *s*, define

(60) $$\begin{array}{c} {\overset{-}{\theta }}_{i}=\{\begin{array}{cc} \mu_{i}\theta_{i}^{*}, & \text{if}\;\;i\in I,\\ \theta_{i}^{*}, & \text{if}\;\;i\notin I, \end{array}\\ {\overset{-}{\varphi }}_{r}=\{\begin{array}{cc} \eta_{r}\varphi_{r}^{*}, & \text{if}\;\;r\in O,\\ \varphi_{r}^{*}, & \text{if}\;\;r\notin O. \end{array} \end{array}$$

By (54) and (56)–(59), $\left(\tilde{\lambda },\overset{-}{\theta },\overset{-}{\varphi }\right)$ is a feasible solution of problem (1) (only restrictions on the right have replaced the *X*_*o*_ and *Y*_*o*_ by ${\overset{-}{\alpha }}_{o}$ and ${\overset{-}{\beta }}_{o}$ in problem (1)) where $\overset{-}{\theta }=({\overset{-}{\theta }}_{1},\ldots ,{\overset{-}{\theta }}_{m})$ and $\overset{-}{\varphi }=({\overset{-}{\varphi }}_{1},\ldots ,{\overset{-}{\varphi }}_{s})$ . Therefore

(61) $$\begin{array}{c} {\overset{-}{\rho }}_{o}=\frac{\left(1/m\right)\sum_{i=1}^{m}{\overset{-}{\theta }}_{i}}{\left(1/s\right)\sum_{r=1}^{s}{\overset{-}{\varphi }}_{r}}<\frac{\left(1/m\right)\sum_{i=1}^{m}\theta_{i}^{*}}{\left(1/s\right)\sum_{r=1}^{s}\varphi_{r}^{*}}=\rho_{o}^{*} \end{array}$$

which is against the assumption that is $\text{eff}({\overset{-}{\alpha }}_{o},{\overset{-}{\beta }}_{o})=\text{eff}(X_{o},Y_{o})=\rho_{o}^{*}$ .

## 5. Illustrative Examples

**Example 1**

Consider a problem of three DMU with two inputs *x*_1_, *x*_2_ and two outputs *y*_1_, *y*_2_. The data of inputs, outputs, and ERM-efficiency score are shown in Table 1.

As can be seen, DMU_*C*_ is an ERM-efficient DMU. If the decision maker is interested in increasing the output vector from *Y*_*C*_ = (5,5) to *β*_*C*_ = (6,6), then, to solve the model (5) by employing the weight-sum method [25], a Pareto solution for this MOLP is generated as *α*_C_* = (1.20,2.40). Based on the model (6), *K*_C_* = 1, eff(*X*_*C*_, *Y*_*C*_) = eff(*α*_C_*, *β*_*C*_) = 1, which means that the ERM-efficiency remains unchanged. Hence, due to Theorem 6, when the outputs of DMU_*A*_ increase to *β*_*C*_ = (6,6) if the decision maker would like to preserve the efficiency score of this DMU, the inputs should increase to *α*_C_* = (1.20,2.40).

Also, if the decision maker is interested in increasing the output vector of *Y*_*C*_ = (5,5) to *β*_*C*_ = (5.5,6), then to solve the model (5) by employing the weight-sum method [25], must the input vector of the *X*_*C*_ = (1,2) increase to *α*_C_* = (1.20,2.40). Based on model (6), since *K*_C_* = 11.5/5 > 1 and *k*_C_* = (6/5,1), we have eff(*X*_*C*_, *Y*_*C*_) ≠ eff(*α*_C_*, *β*_*C*_) = 0.96; therefore, according to part (ii) of Theorem 6, we have eff(*X*_*C*_, *Y*_*C*_) = eff(1.20,2.40,6/5∗5.5,1∗6) = 1. This indicates that new DMU in output component the first to amount “0.5” of the lack produce. In other words, using model (4), we have eff(*X*_*C*_, *Y*_*C*_) = eff(1.20,2.40,6/5∗5.5,1∗6) = 1.

**Example 2**

Consider a problem of four DMU with one input *x* and one output *y*. The data of input, output, and ERM-efficiency scores are shown in Table 2.

As can be seen, DMU_*B*_ is an ERM-efficient DMU. Assume the decision maker identified rate of increase input and output for this DMU, respectively, as 2 ≤ *x* ≤ 7, 4 ≤ *y* ≤ 10. In order to propose patterns to the decision maker to increase input and output for this DMU, under preserving the ERM-efficiency index, based on model (49) the following MOLP model is considered:

(62) min⁡ αBmax⁡ βB s.t.  λ1+2λ2+5λ3+6λ4≤θB∗αB     λ1+4λ2+5λ3+7λ4≥φB∗βB     αB≥2,  βB≥4     αB≤7,  βB≤10     λj≥0, j=1,…,4.

Using the weight-sum method [25], a Pareto solution is generated for this MOLP as (*α*_B_*, *β*_B_*) = (5,10). Therefore, according to Theorem 15, when the inputs and outputs of DMU_*B*_ increase to 5 and 10, respectively, then the ERM-efficiency score of this DMU is 1.

## 6. Conclusion

In the present paper, a typical inverse optimization problem on the nonradial enhanced Russell model has been studied: two main questions on inverse DEA have been discussed and the models have been proposed to estimate the input (output) levels of a given DMU when some or all its output (input) levels were increased under the constant ERM-efficiency score. To determine sufficient and necessary conditions of estimated inputs (outputs), Pareto solutions of the MOLP were used. Moreover, in finding inputs (outputs), if there exists lack output (extra input) in each of a output (input) components, the amount of the lack (extra) is specified by auxiliary models. For an ERM-efficient DMU, necessary and sufficient conditions were introduced to find the minimum and maximum increase of input and output levels, respectively, provided that the ERM-efficiency score remains unchanged. Therefore, the patterns can be presented to the decision maker in order to increase inputs and outputs (extending decision making units) for an ERM-efficient DMU such that the ERM-efficiency remains unchanged. In other words, inverse DEA can be used in theoretical and practical purposes such as strategic planning, management control, resource allocation, and ranking. The sufficient conditions were established only for the ERM-efficient DMU, but the given necessary conditions were for each ERM-efficient or ERM-inefficient DMU. Finding sufficient conditions for ERM-inefficient DMU_*s*_ can be a suitable research field. In addition, using nonradial models under intertemporal dependence, solving the problems introduced by Wei et al. [14], Hadi-Vencheh et al. [16], and the new problem discussed in this paper can be a useful research topic.

## Figures and Tables

**Table 1:** The data and ERM efficiency in Example 1.

DMUs	*x*_1_	*x*_2_	*y*_1_	*y*_2_	*θ*_1_*	*θ*_2_*	*φ*_1_*	*φ*_2_*	ρ*
*A*	4.00	2.00	4.00	4.00	1.00	1.00	4.00	2.00	0.33
*B*	2.00	1.00	8.00	4.00	1.00	1.00	1.00	1.00	1.00
*C*	1.00	2.00	5.00	5.00	1.00	1.00	1.00	1.00	1.00

**Table 2:** The data in Example 2.

DMUs	*x*	*y*	θ*	φ*	ERM efficiency
*A*	1.00	1.00	0.50	1.00	0.50
*B*	2.00	4.00	1.00	1.00	1.00
*C*	5.00	5.00	0.67	1.33	0.50
*D*	6.00	7.00	0.79	1.35	0.58
